# Nursing interventions for improving quality of life among parents with major Thalassemic children: a scoping review

**DOI:** 10.1186/s12912-025-02994-1

**Published:** 2025-03-26

**Authors:** Ai Mardhiyah, Iyus Yosep, Sri Hendrawati, Windy Rakhmawati, Khoirunnisa Khoirunnisa, Theresia Eriyani, Rohman Hikmat

**Affiliations:** 1 Department of Pediatric Nursing, Faculty of Nursing, Universitas Padjadjaran, Sumedang, Jawa Barat Indonesia; 2 Department of Mental Health Nursing, Faculty of Nursing, Universitas Padjadjaran, Sumedang, Jawa Barat, Indonesia; 3 Department of Fundamental Nursing, Faculty of Nursing, Universitas Padjadjaran, Sumedang, Jawa Barat, Indonesia; 4 Nursing Department, Faculty of Health Science, Universitas ‘Aisyiyah Bandung, Bandung, Indonesia

**Keywords:** Children, Intervention, Parents, Quality of life, Thalassemia

## Abstract

**Introduction:**

Thalassemia major is a chronic genetic disease requiring long-term treatment, significantly impacting the quality of life of affected children and their parents. Parents often experience emotional, social, and economic burdens in caring for children with thalassemia. Nursing interventions play a crucial role in improving their quality of life by providing holistic support. This scoping review aims to explore nursing interventions that enhance the quality of life of parents with children diagnosed with thalassemia major.

**Methods:**

A scoping review methodology was applied. Literature searches were conducted in CINAHL, PubMed, and Scopus, as well as Google Scholar. The primary keywords included “nursing intervention,” “quality of life,” “thalassemia,” “parents,” and “family support.” Inclusion criteria comprised full-text, original research articles published in English from 2015 to 2024. Data extraction was performed manually, and qualitative descriptive analysis was used.

**Results:**

Nine studies met the inclusion criteria, discussing various nursing interventions to improve parental quality of life. Effective interventions included positive thinking therapy, family empowerment, counseling, and education, which enhanced parental knowledge, coping skills, emotional well-being, and overall quality of life. Key factors supporting intervention success included active parental participation, continuous healthcare support, and the availability of accessible educational resources.

**Conclusion:**

This study highlights the essential role of nurses in supporting parents of children with thalassemia. A holistic nursing approach is crucial in addressing the multifaceted needs of these parents. Further research should explore the long-term effectiveness of these interventions and their adaptation to individual family circumstances.

## Introduction

Thalassemia is one of the most prevalent hereditary hemolytic diseases worldwide, affecting thousands of children annually due to genetic mutations that disrupt hemoglobin synthesis [1, 2]. Beta thalassemia major is a severe inherited blood disorder characterized by chronic anemia and requiring lifelong medical intervention, such as blood transfusions and iron chelation therapy [3]. This condition imposes a significant burden not only on the affected children but also on their families, particularly their parents.

Globally, the prevalence of beta-thalassemia carriers varies, with rates ranging from 1 to 19% in different populations [4]. Southeast Asia has a carrier rate of approximately 3-9%, with Thailand (3-9%), Malaysia (1-4.5%), and Indonesia (3%) among the most affected regions [3, 5]. Indonesia reports an estimated 2,500 new thalassemia cases annually, with the total number of registered patients increasing from 4,896 in 2012 to 9,028 in 2018 [6].

Parents of children with thalassemia experience significant emotional, social, and financial stress. Their understanding of the disease encompassing its nature, progression, and management shapes their emotional and behavioral responses, which in turn affect their overall well-being [7]. Several studies have documented that parents of children with chronic illnesses report a lower quality of life due to psychological distress, economic burden, and limited support systems [8, 9]. A study in India found that parents of children receiving routine transfusion therapy exhibited significantly lower quality-of-life scores compared to those with healthy children [10].

Parental caregiving for children with chronic illnesses demands constant physical and emotional endurance. Mothers, in particular, often report higher levels of stress, anxiety, and depressive symptoms due to their caregiving roles [11]. Key factors contributing to poor parental quality of life include a lack of family-centered care, inadequate psychosocial support, and insufficient disease-related education [12]. A study reported that 67.5% of parents of children with thalassemia experience psychological distress due to the extensive treatment burden and caregiving challenges [13].

The multifaceted impact of thalassemia on parental quality of life, emphasizing both the psychological and socioeconomic challenges faced by caregivers. Research conducted in Iran found that parents of children with thalassemia major experience significantly higher levels of anxiety and depression compared to parents of healthy children, largely due to the chronic nature of the disease and the frequent need for hospital visits and medical procedures [12, 14]. Parents of children with thalassemia suffer from moderate to severe psychological distress, with financial constraints being a major contributing factor [15]. In Indonesia, caregivers of children with thalassemia frequently express concerns about the long-term economic burden, as the costs associated with lifelong blood transfusions and iron chelation therapy often surpass their financial capabilities [8, 16]. Additionally, qualitative studies indicate that parents face substantial social isolation, as the time-consuming demands of caregiving limit their ability to participate in social and recreational activities, further exacerbating feelings of distress and fatigue [17, 18].

Interventions designed to enhance the quality of life of parents with chronically ill children have been shown to improve coping strategies, psychological resilience, and caregiving efficiency [19]. Studies indicate that improving parental quality of life indirectly benefits the child, as better parental mental well-being translates into improved caregiving practices [20]. Nurses play a critical role in addressing the psychosocial needs of parents through various interventions, such as psychoeducation, cognitive-behavioral strategies, and support group facilitation [21].

Although previous reviews have focused on interventions to improve the quality of life of children with thalassemia, there is a lack of comprehensive literature examining interventions specifically targeting parental quality of life [22, 23]. Given the significant impact of caregiving stress on parents and its indirect effect on child outcomes, this study aims to fill the gap by reviewing nursing interventions that enhance the quality of life of parents with children diagnosed with thalassemia major. This scoping review aims to explore nursing interventions that enhance the quality of life of parents with children diagnosed with thalassemia major.

## Methods

### Study design

This study utilized a scoping review design based on the Arksey and O’Malley framework [24]. The purpose of this design was to map the existing literature on interventions aimed at improving the quality of life of parents with a child with thalassemia. This design was selected to assess the breadth of the literature, identify research gaps, and summarize findings from diverse studies. The five key stages of the scoping review included: (1) identifying the research question, (2) identifying relevant studies, (3) study selection, (4) data charting, and (5) collating, summarizing, and reporting results. This approach provided a systematic yet flexible method for exploring and synthesizing various types of research evidence.

### Search strategy and eligibility criteria

The literature search was conducted in Scopus, PubMed, and CINAHL databases, along with the Google Scholar search engine. These databases were chosen due to their extensive coverage and relevance in the fields of health and nursing. The search strategy used Boolean operators and MeSH terms, with keywords including “nursing intervention,” “quality of life,” “parents,” “children,” and “thalassemia.” The research question was: “What are the interventions implemented to improve the quality of life of parents with a child with thalassemia, and what research gaps exist?” The search was conducted between January and March 2024 to ensure the inclusion of the most recent studies.

The search queries applied in different databases were as follows:

Scopus: TITLE-ABS-KEY(“nursing intervention” OR “nursing care” OR “nursing practice” OR “nursing management”) AND TITLE-ABS-KEY(“quality of life” OR “well-being” OR “health-related quality of life” OR “HRQoL”) AND TITLE-ABS-KEY(“parents” OR “caregivers” OR “family members” OR “mother” OR “father”) AND TITLE-ABS-KEY(“children” OR “child” OR “pediatrics” OR “adolescents” OR “youth”) AND TITLE-ABS-KEY(“thalassemia” OR “beta-thalassemia” OR “thalassemia major” OR “blood disorder”).

PubMed: ((“nursing intervention“[MeSH] OR “nursing care“[MeSH] OR “nursing practice” OR “nursing management”) AND (“quality of life“[MeSH] OR “well-being” OR “health-related quality of life” OR “HRQoL”) AND (“parents“[MeSH] OR “caregivers“[MeSH] OR “family members” OR “mother” OR “father”) AND (“children“[MeSH] OR “child“[MeSH] OR “pediatrics“[MeSH] OR “adolescents“[MeSH] OR “youth”) AND (“thalassemia“[MeSH] OR “beta-thalassemia” OR “thalassemia major” OR “blood disorder”))

CINAHL: (“nursing intervention” OR “nursing care” OR “nursing practice” OR “nursing management”) AND (“quality of life” OR “well-being” OR “health-related quality of life” OR “HRQoL”) AND (“parents” OR “caregivers” OR “family members” OR “mother” OR “father”) AND (“children” OR “child” OR “pediatrics” OR “adolescents” OR “youth”) AND (“thalassemia” OR “beta-thalassemia” OR “thalassemia major” OR “blood disorder”).

Google Scholar: (“nursing intervention” OR “nursing care” OR “nursing practice” OR “nursing management”) AND (“quality of life” OR “well-being” OR “health-related quality of life” OR “HRQoL”) AND (“parents” OR “caregivers” OR “family members” OR “mother” OR “father”) AND (“children” OR “child” OR “pediatrics” OR “adolescents” OR “youth”) AND (“thalassemia” OR “beta-thalassemia” OR “thalassemia major” OR “blood disorder”).

**Fig. 1:**
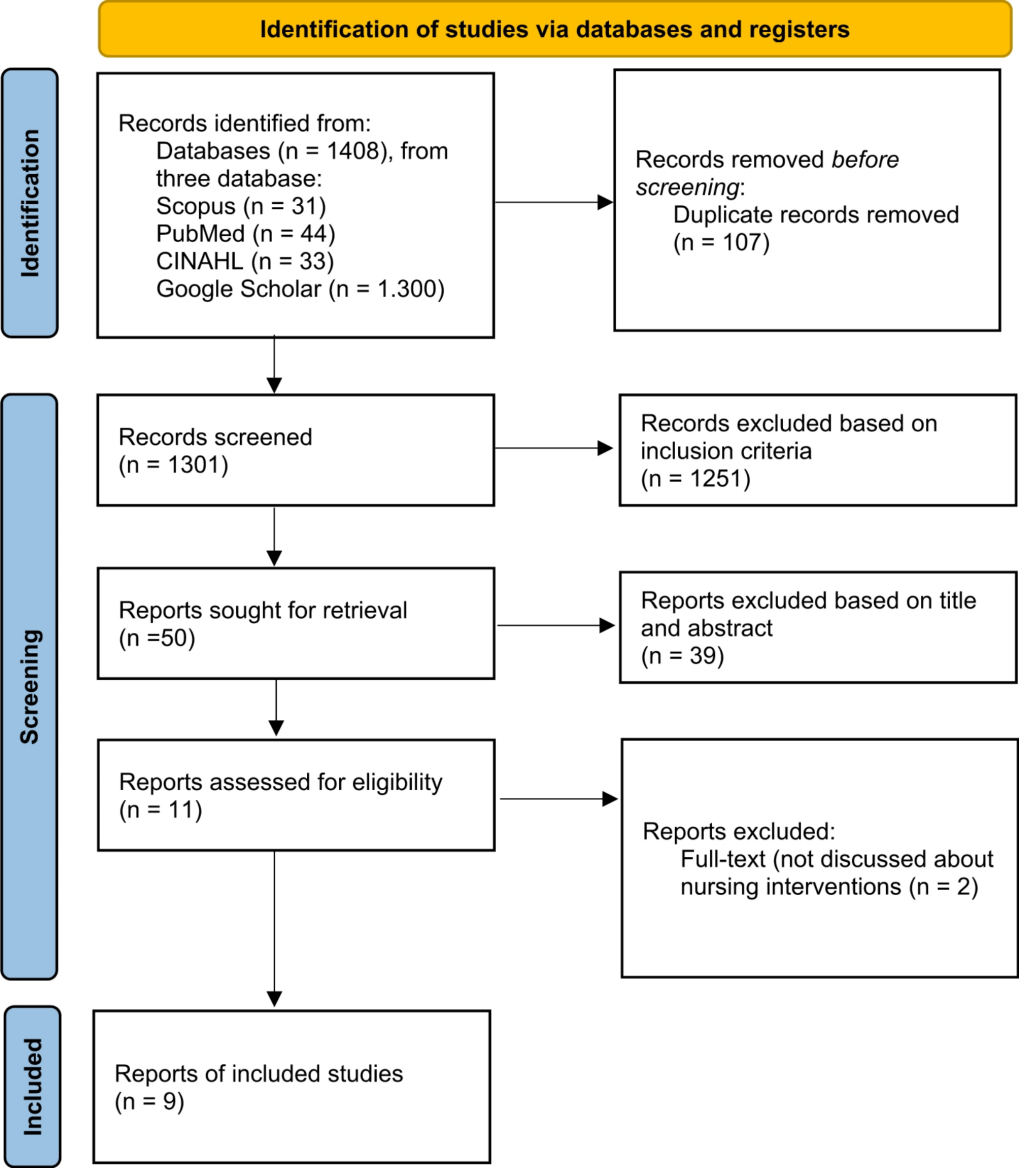
PRISMA flow diagram

The inclusion criteria for this study were determined using the PCC (Population, Concept, Context) framework. The population consisted of parents with a child diagnosed with thalassemia. The concept focused on nursing interventions aimed at improving the quality of life of these parents, while the context included studies examining the impact of such interventions on quality of life outcomes. Both qualitative and quantitative studies were included, encompassing randomized controlled trials (RCTs), cohort studies, cross-sectional studies, and qualitative research. To ensure relevance to current nursing practice, only articles published in English within the last ten years (2015–2024) were considered. Exclusion criteria included articles available only as abstracts or grey literature and studies that focused solely on the medical treatment of thalassemia without addressing parental quality of life. The study selection process was documented using a PRISMA flow diagram (Fig. 1).

### Data extraction

Data extraction was performed manually using Microsoft Excel and included the following categories: author, purpose, study design, sample, country, intervention type, outcome measures, and quality-of-life assessment tools. A critical aspect of the extraction was identifying the types of instruments used to measure quality of life, as this can vary between global quality-of-life assessments and health-related quality of life (HRQoL) scales. Two authors conducted the extraction independently to enhance accuracy. Any disagreements were resolved through discussion, and if necessary, a third author with expertise in the field was consulted to reach a final consensus.

### Quality appraisal

The quality assessment of the articles was analyzed using the critical appraisal method from The Joanna Briggs Institute (JBI). JBI is a critical appraisal tool that evaluates the quality of articles based on specific criteria to determine their eligibility for review. The number of assessment statements for quasi-experimental studies is nine, case study design have 8 statements, while randomized controlled trials are evaluated using 13 statements. Each JBI assessment statement is scored as “yes,” “no,” “unclear,” or “not applicable.” A score of “yes” is assigned a value of 1, while all other responses are given a score of zero. Articles considered suitable for analysis in this study were those with a score above 75%, based on the criteria and relevance to the topic (Table 1).

**Table 1:** JBI critical appraisal tool

Author, published year	JBI critical appraisal Tool	Study design
(Sadeghloo et al. [19])	88,9% (8/9)	Quasi experimental
(Wacharasin et al. [27])	100% (9/9)	Quasi experimental
(Zareban et al. [33])	92,3% (12/13)	RCT
(Mariani et al. [29])	77.8% (8/9)	Quasi experimental
(Lotfi et al. [32])	88,9% (8/9)	Quasi experimental
(Firmansyah et al. [28])	87,5% (7/8)	Case study
(Setiawan et al. [31])	87,5% (7/8)	Case study
(Mahmoud & Yossif, [30])	88,9% (8/9)	Quasi experimental

### Data analysis

A descriptive qualitative approach was employed for data analysis, utilizing thematic analysis to identify patterns across studies. The analysis followed Braun and Clarke (2013) thematic framework, which involved several steps: familiarization with the data, generating initial codes, searching for themes, reviewing themes, defining and naming themes, and producing the final thematic synthesis [26]. To ensure objectivity and depth, two authors independently coded the data. In cases of discrepancies, a third author was consulted to finalize the categorization. This method allowed for a comprehensive synthesis of various interventions and their impact on parental quality of life.

## Results

Based on the initial research, there were 1408 reports, 108 reports from three databases: CINAHL, PubMed, and Scopus and from search engine we found 1300 reports from google scholar. The authors eliminated 107 duplicate articles. Then, the authors eliminated 1251 articles that did not meet the inclusion criteria based on the research objective. Next, the authors eliminated 39 articles that were not relevant based on the title and abstract. After that, the authors read the full-text of the remaining articles and found 9 articles that discussed interventions to improve the quality of life of parents with a child with thalassemia.

This scoping review analyzed a total of nine articles that discussed interventions to improve the quality of life of parents with a child with thalassemia. The research designs of these articles included six quasi-experimental studies, one randomized controlled trial (RCT), and two case studies. The articles were from four countries: Iran, Indonesia, Thailand, and Egypt, with three articles from Iran, three from Indonesia, one from Thailand, and two from Egypt. The number of samples involved in these articles varied, ranging from 2 to 71 participants.

The results of this scoping review showed that there are four types of interventions that can be done to improve the quality of life of parents with a child with thalassemia: positive thinking therapy, family empowerment, counseling, and education. The data results can be seen in Table 2 (Table 2):

**Table 2:** Extraction data

Authors, year	Aims	Country	Sample size	Study design	Role of nurses	Interventions	Results
(Sadeghloo et al. [19])	Determining the effect of positive thinking training on the quality of life of parents of adolescents with thalassemia	Iran	52 parents of adolescents with thalassemia	Quasi experimental	Positive thinking training for parents of children with thalassemia to improve their quality of life. This program consists of sessions that foster an optimistic mindset and help parents manage stress and anxiety related to their child’s condition.	The positive thinking training was conducted by researchers with a Master’s degree in pediatric nursing and their supervisor with a PhD in psychiatric nursing over 10 sessions (45–60 min per session) for 6 consecutive weeks in the hospital’s education room.	There was a significant difference in the quality of life scores in the intervention group before and after the intervention (*p* < 0.001)
(Wacharasin et al. [27])	To examine the effectiveness of a Family Empowerment Program (FEP) on family function, family empowerment, and quality of life for families with a child with thalassemia.	Thailand	56 families	Quasi experimental	A family empowerment program to enhance family functioning and well-being in caring for children with thalassemia. This intervention helps families understand their roles in managing their child’s disease and improves their caregiving skills.	The participants received the Family Empowerment Program (FEP) and standard care, divided into three groups with 10 families per group (12–15 participants per group), and the FEP was conducted over four days.	There was a significant improvement in the scores for family function, quality of life, and empowerment over time.
(Zareban et al. [33])	To evaluate the effectiveness of implementing an educational program in adopting thalassemia prevention behaviors and improving quality of life.	Iran	71 participants	RCT	An educational program based on the Theory of Planned Behavior (TPB) to enhance preventive behaviors among mothers of children with thalassemia. Nurses teach healthy behaviors, enhance mothers’ self-control, and provide support in decision-making related to prevention.	The educational method used in this study was a 20-minute educational video about thalassemia, covering topics such as safe pregnancy prevention methods, the importance of prenatal diagnosis, and the social, economic, and psychological consequences for couples and families.	There was a significant increase in perceived behavior, behavioral intention, quality of life, and actual behavior in the intervention group after the intervention (*P* < 0.05).
(Mariani et al. [29])	To determine the influence of family psycho-educational intervention on coping strategies and quality of life among parents caring for a child with thalassemia.	Indonesia	44 parents	Quasi experimental	Psychoeducation intervention for parents of children with thalassemia to improve their coping strategies. Nurses provide psychosocial support and guide parents in dealing with emotional and social challenges in caring for their children.	After the first session, which involved providing educational information about thalassemia, the parents were given a 15-minute break, and then the second session commenced. The second session focused on the parents sharing coping strategies among themselves to manage the stress associated with caring for a child with thalassemia.	Significant improvement in the quality of life of parents.
(Lotfi et al. [32])	To evaluate the impact of a care program using the teach-back method on the knowledge, attitudes, quality of life, and performance of parents of children with thalassemia.	Iran	62 parents of children with β-thalassemia.	Quasi experimental	Facilitators in the teach-back program aimed at improving parents’ knowledge, attitudes, and performance in caring for children with thalassemia.	The training program was implemented individually during morning shifts from April 2022 to February 2023, with the number of training sessions for each parent varying between 3 and 6 sessions (60–90 min) depending on the parent’s learning, patience, and availability.	There was a significant difference in the scores of knowledge, attitudes, and quality of life.
(Firmansyah et al. [28])	To evaluate the Family Empowerment Program (FEP) on the quality of life and care methods of parents with children with thalassemia.	Indonesia	2 participants	Case study	Helping families implement the Family Empowerment Program (FEP), which aims to improve the quality of life of children with thalassemia through education and family support. Nurses assess family needs, provide training, and evaluate changes after the intervention.	The Family Empowerment Program (FEP) was used as an approach to care for children with thalassemia and their quality-of-life issues.	The family empowerment approach significantly improved social functioning and quality of life.
(Setiawan et al. [31])	To measure the level of usefulness of the Cyber Gen application as a medium for indirect genetic counseling for parents with children with thalassemia.	Indonesia	30 participants	Case study	Using the Cyber Gen mobile application to provide genetic counseling services to thalassemia patients and caregivers. This application helps deliver basic information, consultations, and social support, making technology-based nursing services more accessible.	The counselors or consultants include registered nurses who are members of the Indonesian National Nurses Association and have attended training clinics for the practice of genetic counseling in the nursing profession.	The Cyber Gen application can be used to provide genetic counseling interventions to thalassemia patients and caregivers to improve their quality of life.
(Mahmoud & Yossif [30])	To evaluate the influence of a counseling program for mothers to improve the quality of life of parents with children who have thalassemia.	Egypt	51 mothers	Quasi experimental	Providing counseling to mothers of children with thalassemia, helping them better understand their child’s disease and improve their child’s quality of life. The intervention includes educational sessions on disease management and skill enhancement for mothers in caring for their children.	The counseling program was implemented over a 6-month period, consisting of 5 sessions, with each session ranging from 21 to 31 min in duration. The sessions were conducted either individually or in groups of 2 to 4 mothers and their children in the waiting room of the polyclinic.	Counseling can improve knowledge and quality of life.
(Rakhmilla et al. [34])	To evaluate an educational intervention to improve the knowledge, management, and quality of life of mothers with children who have β-thalassemia.	Egypt	38 participants	Quasi experimental	Educating high school students about thalassemia prevention using various methods such as conventional teaching, animated videos, and peer education.	Educational intervention on diet is very important because β-thalassemia causes an increase in iron absorption in the digestive system, leading to iron overload. Therefore, patients are advised to avoid foods that are high in iron, such as liver, spleen, cereals, nuts, leafy green vegetables, and molasses.	Social and professional support are highly recommended for the effectiveness of care in improving quality of life.

### Positive thinking training

The positive thinking training was conducted by researchers with a Master’s degree in pediatric nursing and supervised by those with a PhD in psychiatric nursing. The training consisted of 10 sessions (45–60 min per session) carried out over 6 consecutive weeks in the hospital’s education room [17]. Each session started by introducing the parents and their problems, followed by educational content, and a new discussion. The parents were asked to practice and apply the topics learned. A paired t-test showed a significant difference in quality of life scores in the intervention group before and after the intervention (*p* < 0.001), while there was no significant difference in the control group (*p* = 0.11). An analysis of covariance showed a significant difference between the intervention and control groups in terms of quality of life scores (*p* = 0.009, η = 0.13), indicating that 13% of the change after the intervention was due to the intervention itself.

### Family empowerment program

The Family Empowerment Program (FEP) was implemented by dividing the participants into three groups with 10 families per group [27]. The FEP was conducted over four days with two separate two-day sessions over two weeks. The principal investigator and research associates who carried out the FEP were doctoral-trained nurses [28]. The program was designed to improve family function and empowerment by considering family dynamics and the influence of the illness [29]. T-tests and analysis of variance showed that family caregivers who participated in the FEP had a significant increase in family function and empowerment scores over time.

### Counseling

Cyber Gen Genetic Counseling was conducted by registered nurses who followed the genetic counseling practice clinic. The Cyber Gen application could be used to provide genetic counseling interventions to thalassemia patients and caregivers [30]. The Traditional Counseling Program was carried out over 6 months with 5 sessions (21–31 min per session), held individually or in groups in the outpatient clinic waiting room [31]. Teaching methods included small group discussions, brainstorming, demonstrations, and visual aids such as brochures and posters [32]. The counseling program improved mothers’ knowledge and practices in caring for children with thalassemia, as well as enhancing the quality of life of the thalassemia children.

### Education

A 20-minute educational video on thalassemia, followed by a discussion and Q&A session, as well as the distribution of educational pamphlets. The thalassemia couples filled out the questionnaire again after one month [33]. The average scores of perceived behavioral control, behavioral intention, and behavior in the intervention group increased significantly after the intervention (*p* < 0.05).

Psychoeducation with a Digital Pocketbook consisted of two sessions [34]. First session provided educational information about thalassemia, followed by second session which focused on coping strategies for managing stress, and ended with the distribution of a digital pocketbook via WhatsApp. There was a significant difference in the average scores in the intervention group with a p-value of 0.000, while in the control group, there was no significant difference (p-value 0.492). The difference in average scores between the intervention and control groups was significant (p-value 0.023).

Education on the importance of diet to avoid high-iron foods such as liver, spleen, cereals, nuts, leafy greens, and molasses. Dietary education requires ongoing support, and premarital counseling is necessary, as well as social and professional support, which are highly recommended.

### Role of nurses

Nurses play a vital role in various interventions to improve the understanding, skills, and well-being of patients and families facing thalassemia. One of the main roles of nurses is to provide thalassemia prevention education to high school students through conventional methods, animated videos, and peer education to increase their awareness [34]​. In addition, nurses also act as facilitators in teach-back programs to improve parents’ knowledge and skills in caring for children with thalassemia through effective communication [32].

Psychoeducational interventions are provided to parents to help them improve coping strategies and face the emotional and social challenges of caring for a child with thalassemia [29]. In addition, nurses support family empowerment through the Family Empowerment Program (FEP), which aims to improve the quality of life of children with thalassemia through education and family support [28]. Counseling is also provided to mothers of children with thalassemia to help them understand their child’s disease and improve their quality of life through education and strengthening of care skills [30].

Educational interventions based on the Theory of Planned Behavior (TPB) were implemented to improve thalassemia prevention behavior in mothers by strengthening healthy behavior and self-control in decision making [33]. In addition, nurses provide positive thinking training to parents to improve their quality of life, by building an optimistic mindset and helping them manage stress and anxiety [17]. Nurses can utilize the Cyber ​​Gen application to provide genetic counseling services to thalassemia patients and caregivers, which include basic information, consultation, and technology-based social support [31]. Nurses also implement family empowerment programs to improve family function and well-being in caring for children with thalassemia, helping them understand their role in disease management and improving care skills [27].

### Scientific gap

Based on the analysis of nine articles investigating various interventions to improve the quality of life of thalassemia patients and their families, several scientific gaps can be identified. While various interventions such as positive thinking training, family empowerment programs, counseling, and dietary education have been implemented, no studies have comprehensively integrated a holistic approach that simultaneously addresses psychological, social, educational, and nutritional aspects to achieve long-term improvements in the quality of life for patients and their families. The duration and frequency of interventions also vary significantly, ranging from 4 days to 6 months with sessions lasting between 20 and 90 min, with no consensus on the optimal duration or frequency to achieve maximum outcomes. Most studies also focus only on evaluating the short-term effects of interventions, leaving limited information about the sustainability of quality-of-life improvements beyond six months.

The use of digital technology in supporting interventions remains minimal, with only one article highlighting a technology-based application, such as Cyber Gen. This indicates a significant opportunity to develop more extensive and sustainable technology-based interventions, especially in the digital era. Furthermore, although some interventions are conducted individually, approaches that are genuinely tailored to the specific needs of patients or families—such as considering age, education level, or socioeconomic conditions—are rarely found. These gaps emphasize the need for further research to develop more integrated, personalized, and evidence-based interventions to holistically and sustainably support the quality of life of thalassemia patients and their families.

## Discussion

The results of this study show that various interventions have a positive impact in improving the quality of life of parents with children with thalassemia. Positive thinking training, family empowerment programs, counseling, and education all showed significant improvements in certain aspects of quality of life, family function, and empowerment. Positive thinking training has been shown to improve the quality of life of parents by providing them with tools to cope with the stress and anxiety they experience [35]. The Family Empowerment Program (FEP) successfully increased family function and empowerment through improved understanding and dynamic support for the thalassemia disease. Counseling, both through digital applications and traditional methods, provided improvements in thalassemia care knowledge and practices [36]. Education, including through videos, psychoeducation, and dietary interventions, showed improvements in health behaviors and the quality of life of both children and parents. Overall, these various interventions demonstrate the positive impact they can have in enhancing the quality of life for parents caring for children with thalassemia.

This research is in line with previous studies that have shown that psychosocial support and education play an important role in improving the quality of life of patients and families with chronic conditions [37]. Other research has found that psychosocial interventions significantly improve the quality of life of patients with chronic diseases [38]. A study by Boonchooduang et al. (2015) found that education and family support can improve health outcomes for cancer patients. This indicates that parents with children who have chronic illnesses require psychosocial support to enhance their own quality of life. However, it is important to note that some studies included both parents, while others focused solely on mothers. The findings suggest that interventions targeting both parents tend to yield higher improvements in family function and empowerment, whereas mother-focused interventions primarily impact maternal emotional well-being and coping mechanisms. This distinction highlights the importance of involving both parents in interventions to optimize family support structures.

The role of nurses in interventions to improve the quality of life of parents with children with thalassemia is crucial. Nurses serve as facilitators, educators, and supporters who help parents understand and manage their child’s health condition. In positive thinking training, nurses provide educational sessions and practical exercises that help parents cope with stress and anxiety. The Family Empowerment Program (FEP) guided by nurses enhances family function and empowerment through a structured approach. Nurses also provide genetic counseling and traditional counseling programs that help increase knowledge and improve thalassemia care practices [40]. Through education, whether through videos, psychoeducation, or dietary interventions, nurses help parents understand the importance of proper diet and correct care practices, which in turn improves the quality of life of their children [14].

Additionally, the effectiveness of these interventions may be influenced by the age of the children. The reviewed studies encompassed children of different developmental stages, from infants to adolescents. Parents of younger children, such as infants and toddlers, benefited more from educational and dietary interventions due to their direct impact on nutrition and growth. In contrast, parents of school-age children and adolescents found psychosocial interventions, such as counseling and family empowerment programs, to be particularly beneficial as they provided coping strategies and emotional support to navigate the challenges associated with chronic illness. This highlights the need for age-specific interventions to ensure optimal outcomes for both parents and children.

The research findings show that positive thinking training significantly improved the quality of life of parents with children with thalassemia, as indicated by the increased quality of life scores in the intervention group (*p* < 0.001). The ability of positive thinking therapy to help parents cope with the stress and anxiety associated with caring for a child with a chronic condition is noteworthy. Positive thinking is important because it helps individuals focus on aspects that can be controlled and enhances a sense of optimism, which in turn strengthens emotional and mental well-being [41]. These results are consistent with previous research that found psychosocial interventions can significantly improve the quality of life of patients with chronic diseases [42]. Factors contributing to the success of this intervention include the active engagement of participants in therapy sessions, ongoing support from healthcare providers, and the facilitators’ skills in delivering relevant and applicable content [14].

The research findings show that the Family Empowerment Program (FEP) significantly improved family functioning and empowerment of parents with children with thalassemia, with significant increases in family functioning and empowerment scores over time. FEP provided support, education, and coping strategies that helped families better understand and manage their child’s health condition effectively [43]. Family empowerment is important because the family is the primary support unit that plays a crucial role in the long-term care of a child with a chronic condition [44]. These findings are consistent with previous research showing that family education and support can improve cancer patients’ health outcomes [45]. Factors contributing to the success of this intervention include active family involvement in the program, ongoing support from healthcare providers, and a structured approach focused on the family’s needs and dynamics [46]. Additionally, the facilitators’ skills and competence in delivering the content, as well as their ability to create a supportive environment, are crucial for the effectiveness of the family empowerment program [44].

The research findings show that counseling through digital applications as well as traditional methods significantly increased parental knowledge, caregiving practices, and quality of life. Counseling provided relevant information, emotional support, and coping strategies that parents could access and apply in their daily caregiving. Counseling is crucial as it offers ongoing support and helps parents overcome the emotional and practical challenges in caring for a child with a chronic condition [36]. These findings are consistent with previous research that found genetic counseling significantly improved family understanding and management of genetic conditions [47]. Factors contributing to the success of counseling interventions include active participant engagement, the skills and competence of the counselors, and the use of methods that align with the needs and preferences of individuals or groups [48]. Additionally, the accessibility of counseling, whether through digital technology or in-person, plays an important role in ensuring that the information and support provided can reach all the families in need [49].

The educational interventions through educational videos, digital psychoeducation with pocket books, and dietary interventions significantly increased parental knowledge, health behaviors, and quality of life. Education provided accurate and practical information that could help parents better understand and manage their child’s health condition [36]. These findings are consistent with previous research that found health education significantly improved medication adherence and the management of chronic diseases [50]. Factors contributing to the success of educational interventions include active participant engagement, the relevance and quality of the educational materials, and the use of engaging and accessible delivery methods [51]. Additionally, the ongoing support from healthcare professionals and reinforcement through educational aids, such as pamphlets and digital pocket books, also play a crucial role in ensuring that the knowledge provided can be effectively applied by parents in their daily caregiving [52].

The research findings confirm that interventions encompassing positive thinking therapy, family empowerment, counseling, and education have a significant positive impact on the quality of life of parents with thalassemic children. These findings not only demonstrate an increase in parental knowledge and practical skills in caring for their children, but also reveal a tangible improvement in the psychosocial aspects and overall family well-being [53, 54]. This provides a strong foundation for developing and implementing holistic and integrated interventions to support families living with chronic medical conditions like thalassemia.

## Conclusion

This scoping review identifies nine studies focusing on interventions to improve the quality of life of parents with thalassemic children. The review highlights four key intervention types: positive thinking training, family empowerment, counseling, and education. The findings indicate that these interventions significantly enhance parental knowledge, caregiving skills, emotional well-being, and overall quality of life. Each intervention plays a distinct role in helping parents manage their children’s health conditions more effectively. The success of these interventions depends on active participant engagement, continuous support from healthcare providers, and the accessibility and quality of educational materials.

From a practical perspective, the application of these interventions in medical institutions could provide significant benefits, particularly in terms of cost-effectiveness. Implementing structured psychosocial support programs can reduce the burden on healthcare services by improving parental competence in managing thalassemia care at home, thereby minimizing unnecessary hospital visits and complications. Additionally, integrating these interventions into standard healthcare services can enhance family well-being and reduce long-term economic and psychological strain on both families and society.

The findings of this review suggest that nurses should adopt a holistic approach, incorporating psychological therapy, family empowerment, counseling, and education to support families of children with thalassemia. Professional development programs for nurses should include these competencies to ensure effective and sustainable intervention delivery. Future research should explore the long-term impact of these interventions, their cost-effectiveness, and how they can be adapted to various cultural, healthcare, and social contexts. Further studies should also examine the specific needs of families with children of different age groups, as the challenges faced by parents may vary depending on whether their child is an infant, toddler, school-aged, or adolescent.

## Data Availability

All data generated or analysed during this study are included in this published article.
